# Anaplastic Thyroid Carcinoma and High-Grade Tall Cell Papillary Thyroid Carcinoma: Case Report of a Rare Association

**DOI:** 10.3390/reports9010056

**Published:** 2026-02-10

**Authors:** Catalin-Bogdan Satala, Alina-Mihaela Gurau, Gabriela Patrichi, Daniela Mihalache

**Affiliations:** 1 “Dunărea de Jos” University of Galati, Faculty of Medicine and Pharmacy, Medical and Pharmaceutical Research Center, 800008 Galati, Romania; stlcatalin92@yahoo.com (C.-B.S.); daniela.mihalache@ugal.ro (D.M.); 2 Department of Pathology, Clinical County Emergency Hospital Braila, 810325 Braila, Romania; 3 The School for Doctoral Studies in Biomedical Sciences, “Dunărea de Jos” University of Galați, 800008 Galati, Romania; g.alinaaa96@yahoo.com; 4 The Doctoral School of Medicine and Pharmacy, “George Emil Palade” University of Medicine, Pharmacy, Science and Technology, 540142 Targu Mures, Romania

**Keywords:** papillary thyroid carcinoma, tall cell carcinoma, anaplastic thyroid carcinoma, high-grade features

## Abstract

**Background and Clinical Significance**: Anaplastic thyroid carcinoma (ATC) is a highly aggressive malignancy that may arise through dedifferentiation from pre-existing differentiated thyroid carcinomas. The tall cell subtype of papillary thyroid carcinoma (TC-PTC) represents an aggressive variant that has been involved in this tumor progression pathway. **Case Presentation**: We report on a rare case of ATC developed in association with a high-grade TC-PTC. A 67-year-old man presented with an enlarging anterior cervical mass. Imaging identified a suspicious nodule in the right thyroid lobe, and total thyroidectomy was performed. Histologic examination revealed a biphasic tumor composed of a nodular TC-PTC with high-grade features, contiguous with an infiltrative anaplastic carcinoma component. The anaplastic component showed marked pleomorphism, loss of thyroid differentiation markers, and an increased ki67 proliferation index. Multinucleated giant cells exhibited aberrant CD68 expression, without proliferative activity. **Conclusions:** This case illustrates the morphologic association between tall cell papillary thyroid carcinoma with high-grade features and anaplastic thyroid carcinoma, emphasizing diagnostic considerations within the framework of the WHO 5th edition classification.

## 1. Introduction and Clinical Significance

Anaplastic thyroid carcinoma (ATC) is considered one of the most aggressive malignancies, accounting for less than 2% of all thyroid cancers, but responsible for a disproportionate number of thyroid cancer-related deaths [1]. For many years, ATC was regarded as a de novo undifferentiated carcinoma; however, accumulating morphologic and molecular data have demonstrated that a substantial subset of cases develops through progressive dedifferentiation from pre-existing differentiated thyroid carcinomas [2].

Papillary thyroid carcinoma (PTC), the commonest form of differentiated thyroid cancer, encompasses multiple histologic subtypes with distinct clinicopathologic profiles [3]. Among these, the tall cell subtype (TC-PTC) has long been associated with aggressive behavior, including advanced stage at diagnosis, increased recurrence risk, and low survival rates [4]. Despite these well-recognized observations, previous classification systems did not incorporate objective histologic criteria to formally define high-grade behavior within differentiated thyroid carcinomas [3,4].

A significant conceptual shift occurred with the publication of the latest edition of the World Health Organization (WHO) Classification of Tumors of Endocrine Organs, which introduced the category of differentiated high-grade thyroid carcinoma (DHGTC) [5]. This entity is defined by increased mitotic activity and/or tumor necrosis in follicular-derived carcinomas that otherwise retain the architectural and cytologic features of differentiation, providing a framework for recognizing aggressive behavior prior to overt loss of differentiation [6,7].

The coexistence of TC-PTC with high-grade histologic features and ATC within the same lesion is exceedingly rare [4]. This case report documents such an occurrence and underscores the importance of identifying and reporting high-grade histologic features in differentiated thyroid carcinomas, in order to support accurate classification and diagnostic interpretation [8].

## 2. Case Presentation

### 2.1. Clinical History and Presentation

A 67-year-old male patient presented with a progressively enlarging anterior cervical mass, noted over several months, with no significant compressive symptoms, dysphagia, dyspnea, or dysphonia. His medical history was notable for well-controlled arterial hypertension, with no prior history of thyroid disease, radiation exposure to the head and neck region, or autoimmune disorders. The patient reported no personal history of malignancy. Family history was also unremarkable for thyroid cancer or other endocrine neoplasms. On physical examination, a firm, poorly mobile mass was palpated in the anterior cervical region, corresponding to the thyroid gland. No clinically palpable cervical lymphadenopathy was identified. No fine-needle aspiration cytology (FNAC) or fine-needle aspiration biopsy (FNAB) was performed prior to surgery, and the diagnostic evaluation proceeded directly to surgical management based on clinical and imaging findings.

### 2.2. Laboratory Findings

Laboratory investigations revealed thyroid function tests within normal limits, including serum thyroid-stimulating hormone (TSH), free thyroxine (fT4), and triiodothyronine (T3). Serum thyroglobulin levels were not elevated, and anti-thyroid antibodies, including anti-thyroglobulin (anti-TG) and anti-thyroid peroxidase (ATPO) antibodies, were negative. Routine hematologic and biochemical parameters were within normal reference ranges.

### 2.3. Imaging Studies

Thyroid ultrasound demonstrated a large, solid nodule occupying the right thyroid lobe, measuring 5.2 cm in maximum diameter. The lesion was predominantly hypoechoic, with a heterogeneous internal echotexture and irregular, poorly defined margins. Doppler examination revealed increased internal and peripheral vascularity, raising suspicion for malignancy. No suspicious lymph nodes were identified on preoperative imaging. The patient was initially evaluated in another medical center, and only the written ultrasound report was available for review, as the original imaging files were not provided to our institution.

### 2.4. Surgical Findings

The patient subsequently underwent total thyroidectomy. On gross examination, the right thyroid lobe contained a nodular, relatively well-demarcated tumor measuring 5.3 cm in greatest dimension, with a firm, gray-white cut surface. Adjacent to this nodular area, an ill-defined infiltrative component was identified, extending into the surrounding thyroid parenchyma of the right lobe. The tumor was confined to the thyroid gland and did not involve the surgical resection margins. The left thyroid lobe was unremarkable on gross inspection. The surgical procedure and gross examination of the thyroidectomy specimen were performed at the referring institution. The paraffin blocks were submitted to our department for microscopic evaluation and immunohistochemical profiling, in order to confirm the diagnosis and address the differential diagnostic considerations. Consequently, no macroscopic photographs were available, and the gross features were documented exclusively based on the description provided in the original pathology report.

### 2.5. Histopathological Findings

**Figure 1:**
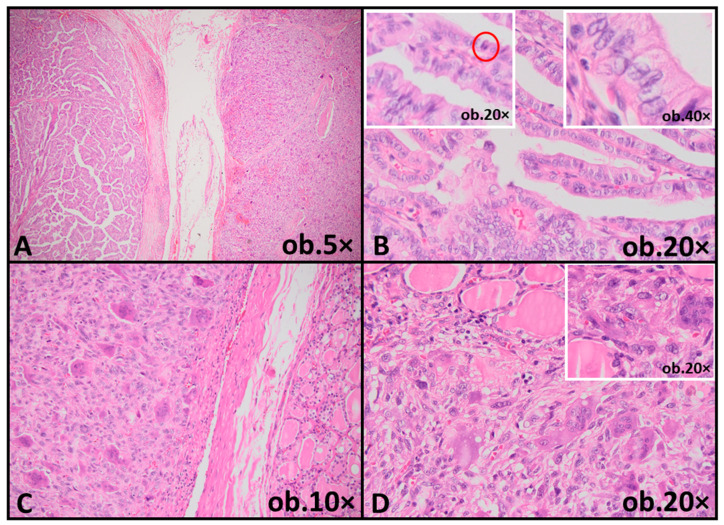
Biphasic thyroid tumor showing a tall cell papillary thyroid carcinoma component and an anaplastic carcinoma component. (**A**) Low-power view illustrating the biphasic architecture of the tumor, with a nodular differentiated component adjacent to a dedifferentiated area. (**B**) Tall cell papillary thyroid carcinoma component composed of slender papillae lined by elongated (right inset), mitotically active tumor cells (left inset, red circle). (**C**) Anaplastic carcinoma component composed of solid sheets of markedly pleomorphic tumor cells and multinucleated giant cells. (**D**) Infiltrative growth of the anaplastic component into adjacent thyroid parenchyma, with residual normal thyroid follicles (inset).

Microscopic examination revealed a biphasic tumor composed of two distinct but contiguous components. The nodular area corresponded to a papillary and trabecular architectural pattern lined by elongated tumor cells measuring at least three times taller than wide. These cells exhibited abundant eosinophilic cytoplasm and classic nuclear features of papillary thyroid carcinoma, including nuclear enlargement, chromatin clearing, nuclear grooves and occasional intranuclear pseudoinclusions. Increased mitotic activity (6 mitoses/2 mm^2^) was identified within this component, consistent with high-grade histologic features. In contrast, the infiltrative component displayed features of anaplastic thyroid carcinoma (ATC). This area was composed of markedly pleomorphic tumor cells arranged in solid sheets, including epithelioid and multinucleated giant cells. Brisk mitotic activity was present (Figure 1).

### 2.6. Immunohistochemical Findings

**Figure 2:**
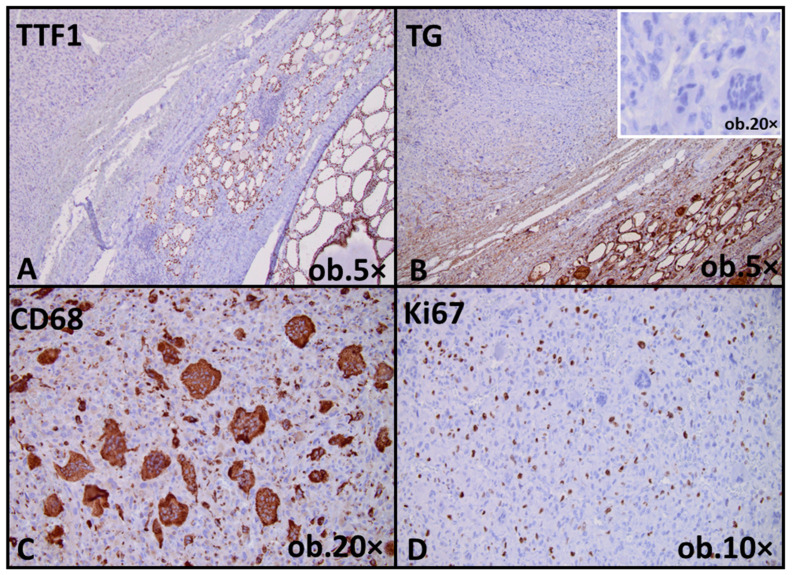
Immunohistochemical profile of the anaplastic component of the tumor. (**A**) Loss of thyroid transcription factor 1 (TTF1) expression in anaplastic tumor cells, with positive endogenous control in adjacent normal follicles. (**B**) Absence of thyroglobulin expression in the anaplastic component (inset), with positive endogenous control in adjacent normal follicles. (**C**) Strong cytoplasmic CD68 immunoreactivity in multinucleated giant cells, reflecting a dedifferentiated phenotype. (**D**) High proliferative activity of anaplastic tumor cells demonstrated by an increased Ki67 labeling index.

The immunohistochemical panel was selected to assess thyroid lineage differentiation, proliferative activity, and potential histiocytic or mesenchymal differentiation, as well as to support the differential diagnosis between differentiated high-grade carcinoma and anaplastic thyroid carcinoma. Immunohistochemical analysis demonstrated loss of thyroid differentiation markers in the anaplastic component, which was negative for thyroglobulin (TG) and thyroid transcription factor 1 (TTF1). Multinucleated giant cells showed strong CD68 immunoreactivity. The Ki67 proliferation index was elevated in the anaplastic component, reaching approximately 25%, while the CD68-positive multinucleated giant cells did not exhibit Ki67 labeling. Overall, the morphologic and immunophenotypic findings supported the diagnosis of anaplastic thyroid carcinoma arising in association with tall cell papillary thyroid carcinoma exhibiting high-grade nuclear features (Figure 2).

### 2.7. Clinical Outcome and Follow-Up

Following surgery, the patient was referred for further oncologic management. Subsequent treatment was performed at a medical center in another country. As the patient was lost to follow-up after leaving the country, no additional clinical, therapeutic, or survival data were available for evaluation.

## 3. Discussion

The tall cell subtype of papillary thyroid carcinoma (TC-PTC) was first recognized in 1976 as a morphologically distinct form of papillary thyroid carcinoma (PTC) [9]. Characterized by elongated tumor cells (at least three times taller than wide), with abundant eosinophilic cytoplasm and nuclei displaying the classical features of PTC, TC-PTC was initially identified primarily on qualitative grounds. Despite these observations, the diagnostic definition of TC-PTC remained inconsistent for decades, with variable thresholds for the proportion of tall cells required for diagnosis and without formal incorporation of histologic grading criteria [9,10]. Over time, larger cohort studies confirmed that TC-PTC represents a biologically aggressive form of differentiated thyroid carcinoma, consistently associated with worse disease-specific outcomes, compared to classic PTC [11]. These findings reinforced the concept that tall cell morphology is not merely a histologic particular aspect, but reflects an underlying aggressive tumor biology. Nevertheless, until recently, mitotic activity and tumor necrosis, features traditionally associated with poorly differentiated thyroid carcinoma (PDTC), were not systematically integrated into the classification of PTC [12].

**Figure 3:**
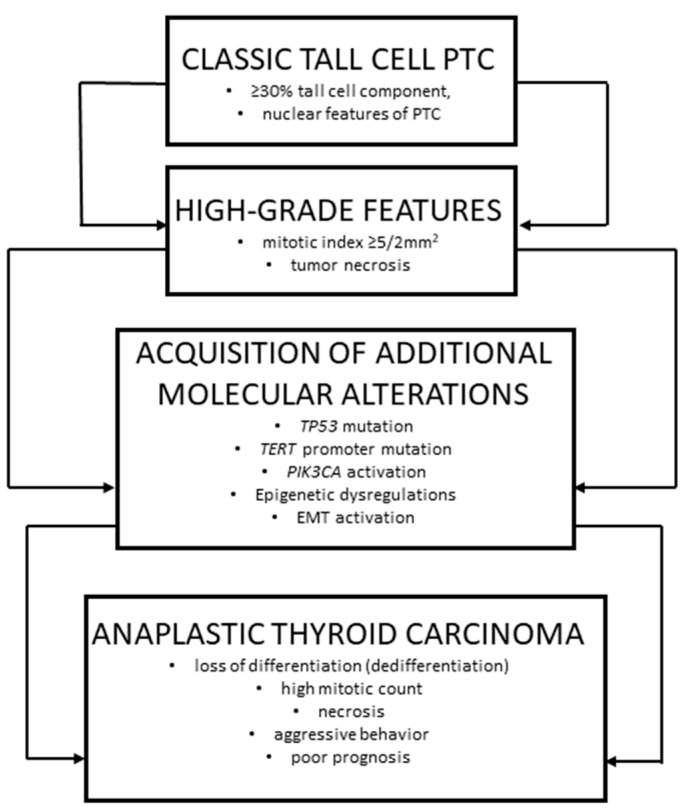
Conceptual schematic representation illustrating a possible morphologic continuum from tall cell papillary thyroid carcinoma to differentiated high-grade thyroid carcinoma and anaplastic thyroid carcinoma, based on histopathological features described in the literature and observed in the present case.

ATC, in contrast, has long been recognized as one of the most aggressive malignancies, characterized by rapid growth, extensive local invasion, early metastasis, and an almost uniformly poor prognosis [13,14,15]. Historically, ATC was considered a de novo undifferentiated carcinoma [16]; however, accumulating morphologic and molecular evidence has demonstrated that a substantial proportion of ATCs arise through dedifferentiation from pre-existing differentiated thyroid carcinomas, including papillary carcinoma, and, notably, the tall cell subtype (Figure 3) [17,18,19,20,21].

The evolution of diagnostic criteria over past decades reflects a gradual recognition of a morphologic and biologic continuum in thyroid tumor progression. The introduction of the Turin Criteria in 2007 provided a framework for identifying PDTC based on architectural patterns and high-grade features, such as increased mitotic activity and tumor necrosis. However, this system excluded papillary carcinomas by definition, leaving a subset of differentiated tumors with high-grade features unclassified from a grading standpoint [22].

In the 4th edition of the World Health Organization (WHO) Classification of Tumors of Endocrine Organs, the tall cell subtype (formerly ‘variant’) was defined as a PTC containing at least 30% tall cells (height at least two–three times larger compared to their width) and exhibiting classic nuclear features of PTC, while ATC and PDTC were recognized as distinct entities [23]. Although this classification acknowledged the aggressive nature of TC-PTC, it did not provide a mechanism for grading differentiated carcinomas based on mitotic activity or necrosis. As a result, papillary carcinomas exhibiting this architecture continued to be classified as differentiated, potentially underestimating their clinical risk.

The 5th edition of the WHO Classification, published in 2022, addressed this limitation through the introduction of differentiated high-grade thyroid carcinoma (DHGTC). This category encompasses follicular-derived tumors, including all their histologic subtypes, that retain architectural and cytologic differentiation but exhibit objective high-grade features, defined as a mitotic count of ≥5/2 mm^2^ and/or the presence of tumor necrosis [24]. Importantly, TC-PTC can now be classified as high grade when these criteria are met, providing a formal diagnostic bridge between differentiated carcinoma and ATC (Table 1).

In current case, the papillary carcinoma component exhibited tall cell morphology accompanied by increased mitotic activity, fulfilling criteria for DHGTC.

An additional noteworthy finding in the anaplastic component of the current case was the presence of multinucleated giant cells showing strong cytoplasmic CD68 immunoreactivity, in the absence of expression of thyroid differentiation markers such as TG and TTF1. Importantly, these CD68-positive giant cells did not exhibit Ki67 labeling, suggesting that they do not represent the actively proliferating tumor population. Similar findings have been described in ATC with giant cell morphology, in which multinucleated tumor cells are considered terminally differentiated or degenerative elements rather than the active clonal proliferative compartment [25,26,27]. Aberrant CD68 expression in this setting is widely regarded as a manifestation of extreme dedifferentiation and phenotypic switch, rather than evidence of true histiocytic differentiation [26]. The absence of proliferative activity within giant cell population, together with the elevated Ki67 index in the remaining anaplastic component, supports a model in which tumor progression is driven by highly proliferative undifferentiated cells, while multinucleated CD68-positive cells reflect end-stage cellular transformation. This immunophenotypic profile further emphasizes the profound loss of thyroid-specific differentiation associated with anaplastic transformation [25,26].

From a diagnostic standpoint, the main differential diagnosis considered in this case was poorly differentiated thyroid carcinoma (PDTC), particularly given the presence of solid growth areas and increased mitotic activity. However, several histologic and immunophenotypic features argued against this diagnosis, including the marked cellular pleomorphism, the prominent multinucleated giant cell component, brisk and atypical mitotic figures, an infiltrative growth pattern, and the complete loss of thyroid differentiation markers, such as thyroglobulin and TTF1. Taken together, these findings are more consistent with an anaplastic thyroid carcinoma, in accordance with the diagnostic criteria outlined in the WHO 2022 classification [24].

An additional diagnostic challenge in the present case was the distinction between differentiated high-grade thyroid carcinoma and early anaplastic transformation, particularly in the context of intratumoral heterogeneity. In this case, the tall cell papillary carcinoma component retained the architectural and nuclear features of differentiation, despite the presence of increased mitotic activity, fulfilling the criteria for differentiated high-grade thyroid carcinoma. In contrast, the adjacent component showed abrupt morphologic transition to marked cytologic pleomorphism, loss of follicular architecture, infiltrative growth, and complete loss of thyroid differentiation markers, supporting a diagnosis of overt anaplastic thyroid carcinoma rather than early or incipient dedifferentiation.

Molecular studies provide further insights into the pathways that may drive this progression. TC-PTC is frequently associated with *BRAF V600E* mutation, which activates the MAPK signaling pathway and contributes to tumor proliferation and aggressiveness [28,29]. As tumor progress, additional genetic alterations, including *TP53* mutations, *TERT* promoter mutations, and *PIK3CA* activation, are frequently acquired, conferring genomic instability and facilitating dedifferentiation [30,31,32]. These alterations are particularly prevalent in ATC and are thought to represent key molecular drivers of progression from differentiated to dedifferentiated tumors. Although these molecular alterations are well documented in the progression from differentiated to anaplastic thyroid carcinoma, molecular genetic testing was not performed in the present case, as the specimen was referred to our institution exclusively for histopathological second opinion and immunohistochemical evaluation.

Beyond genetic changes, epigenetic dysregulation and alterations in the tumor microenvironment may further contribute to tumor evolution [33]. Epigenetic modifications affecting gene expression, dysregulation of cell-cycle checkpoints and activation of epithelial–mesenchymal transition (EMT) have all been involved in the loss of differentiation and acquisition of highly invasive behavior [34,35]. Tumor necrosis, a defining feature of high-grade disease, may also reflect hypoxia and ischemia within the tumor microenvironment, conditions that promote selection of more aggressive tumor clones. From a diagnostic standpoint, the distinction between DHGTC and early anaplastic transformation can be challenging, particularly in limited biopsy material. Tumors may display striking intratumor heterogeneity, with well-differentiated areas co-existing alongside regions of high-grade morphology or overt anaplasia [36]. This underscores the importance of thorough sampling and careful histologic evaluation of thyroidectomy specimens, especially in tumor exhibiting tall cell features.

Clinically, recognition of high-grade features in TC-PTC has important implications for patient management. DHGTC may demonstrate reduced responsiveness to radioactive iodine therapy and may require more aggressive surgical management, closer surveillance, or consideration of alternative therapeutic strategies [37]. Clear reporting of mitotic activity and tumor necrosis facilitates communication between pathologists and clinicians and allows for accurate risk stratification. From a clinical perspective, the recognition of differentiated high-grade thyroid carcinoma has important prognostic and therapeutic implications. Tumors classified as high-grade may exhibit more aggressive behavior, reduced responsiveness to radioactive iodine therapy, and an increased risk of progression compared to conventional differentiated carcinomas. Therefore, accurate identification and reporting of high-grade features may influence clinical decision-making, postoperative surveillance strategies, and consideration of alternative therapeutic approaches.

It is important to emphasize that the concept of a stepwise progression from differentiated to anaplastic thyroid carcinoma remains a theoretical and clinicopathologic construct rather than a universally established biological sequence. The recently introduced category of differentiated high-grade thyroid carcinoma in the WHO 2022 [24] classification provides a diagnostic framework for recognizing aggressive histologic features within otherwise differentiated tumors, but its clinicobiological significance is still being elucidated. In this context, the present case does not aim to establish causality or progression, but rather to illustrate morphologic patterns that are consistent with concepts currently discussed in the literature.

## 4. Conclusions

This case illustrates the rare but significant occurrence of ATC arising in association with a pre-existing TC-PTC with high-grade features. It highlights the importance of recognizing and reporting high-grade histologic features in TC-PTC, as these findings are relevant for accurate classification and diagnostic interpretation within the framework of the WHO 5th edition. The integration of morphologic grading systems, as formalized in the WHO 5th edition, with molecular insights provides a more comprehensive framework for understanding thyroid tumor progression and guiding clinical decision-making. This case report has several limitations that should be acknowledged. These include the unavailability of original ultrasound images and macroscopic photographs of the surgical specimen, as the patient was initially evaluated and surgically treated at another institution, and only descriptive clinical and pathology reports were accessible. In addition, molecular genetic testing was not performed, as the specimen was referred to our institution exclusively for histopathological second opinion and immunohistochemical evaluation. Furthermore, long-term clinical follow-up data were not available, as the patient continued postoperative management in another country and was subsequently lost to follow-up.

## Figures and Tables

**Table 1:** Evolution of histologic grading concepts in follicular-derived thyroid carcinomas, including tall cell papillary thyroid carcinoma and anaplastic thyroid carcinoma.

Period/Classification	Entity	Key Histologic Criteria	Conceptual/Clinical Implications
**1970s—initial descriptions [9]**	Tall cell variant of papillary thyroid carcinoma (PTC)	Tumor cells at least two–three times taller than they are wide; abundant eosinophilic cytoplasm; classic PTC nuclear features; no formal cut-off for proportion of tall cells	First recognition of tall cell morphology; aggressive behavior suspected, but nor formally graded
**1980s–1990s**	Anaplastic thyroid carcinoma (ATC)	Marked cellular pleomorphism; spindle, epithelioid, or giant cells; frequent atypical mitoses; tumor necrosis; complete loss of follicular differentiation	ATC regarded primarily as a de novo undifferentiated carcinoma with uniformly poor prognosis
**1990s–early 2000s**	Tall cell PTC	Variable diagnostic thresholds (30–50% tall cells); absence of standardized grading criteria; mitotic activity and necrosis often noted but not formally integrated	Increasing recognition of aggressive clinical course but persistent diagnostic heterogeneity
**2007—Turin Criteria proposal** **[22]**	Poorly differentiated thyroid carcinoma (PDTC)	Solid, trabecular, or insular growth pattern plus ≥1 of mitotic activity ≥3/10 HPF, tumor necrosis, or convoluted nuclei	Introduced objective high-grade criteria, but excluded PTC with classic papillary architecture
**2017—WHO 4th edition** **[23]**	Tall cell PTC	≥30% tall cells with classic PTC nuclear features; no grading system for high-grade features within PTC	Tall cell PTC formally classified as an aggressive variant, but mitoses and necrosis not used for grading
**2017—WHO 4th edition** **[23]**	ATC	Undifferentiated carcinoma showing spindle, giant, or epithelioid morphology; high mitotic rate; necrosis; invasive growth	Acknowledged that ATC may arise from differentiated carcinomas, but without formal intermediate category
**2022—WHO 5th edition** **[24]**	Differentiated high-grade thyroid carcinoma (DHGTC), including tall cell PTC	Retained differentiated morphology with mitotic activity ≥5/2 mm^2^ and/or tumor necrosis, regardless of architectural pattern	Introduces objective histologic criteria for recognizing high-grade features within otherwise differentiated thyroid carcinomas, providing a standardized diagnostic framework
**2022—WHO 5th edition** **[24]**	Tall cell PTC	≥30% tall cells; three times taller than they are wide; classified as high-grade if mitosis and/or necrosis criteria are met	Allows objective identification of tall cell PTC with increased risk of aggressive behavior
**2022—WHO 5th edition** **[24]**	ATC	Undifferentiated carcinoma, often arising in association with differentiated with/without high-grade component; marked mitoses, necrosis, invasion	Defines ATC as an undifferentiated malignancy that may occur in association with differentiated thyroid carcinomas, acknowledging potential relationships without establishing a defined progression sequence

## Data Availability

The original contributions presented in this study are included in the article. Further inquiries can be directed to the corresponding author.

## References

[1] Juhlin C.C., Mete O., Baloch Z.W. (2022). The 2022 WHO classification of thyroid tumors: Novel concepts in nomenclature and grading. Endocr. Relat. Cancer.

[2] Baloch Z.W., Asa S.L., Barletta J.A., Ghossein R.A., Juhlin C.C., Jung C.K., LiVolsi V.A., Papotti M.G., Sobrinho-Simões M., Tallini G. (2022). Overview of the 2022 WHO Classification of Thyroid Neoplasms. Endocr. Pathol..

[3] Lebrun L., Salmon I. (2024). Pathology and new insights in thyroid neoplasms in the 2022 WHO classification. Curr. Opin. Oncol..

[4] Ghossein R., Katabi N., Dogan S., Shaha A.R., Tuttle R.M., Fagin J.A., Ganly I., Xu B. (2024). Papillary thyroid carcinoma tall cell subtype (PTC-TC) and high-grade differentiated thyroid carcinoma tall cell phenotype (HGDTC-TC) have different clinical behaviour: A retrospective study of 1456 patients. Histopathology.

[5] Jung C.K., Bychkov A., Kakudo K. (2022). Update from the 2022 World Health Organization Classification of Thyroid Tumors: A Standardized Diagnostic Approach. Endocrinol. Metab..

[6] Basolo F., Macerola E., Poma A.M., Torregrossa L. (2023). The 5th edition of WHO classification of tumors of endocrine organs: Changes in the diagnosis of follicular-derived thyroid carcinoma. Endocrine.

[7] Poma A.M., Macerola E., Ghossein R.A., Tallini G., Basolo F. (2024). Prevalence of Differentiated High-Grade Thyroid Carcinoma Among Well-Differentiated Tumors: A Systematic Review and Meta-Analysis. Thyroid.

[8] Goswami P., Patel T., Dave R., Singh G., Singh A., Kalonia T. (2024). WHO 2022 updates on follicular cell and c-cell derived thyroid neoplasm. J. Med. Life.

[9] Hawk W.A., Hazard J.B. (1976). The many appearances of papillary carcinoma of the thyroid. Clevel. Clin. Q..

[10] Morris L.G., Shaha A.R., Tuttle R.M., Sikora A.G., Ganly I. (2010). Tall-cell variant of papillary thyroid carcinoma: A matched-pair analysis of survival. Thyroid.

[11] Ghossein R., Livolsi V.A. (2008). Papillary thyroid carcinoma tall cell variant. Thyroid.

[12] Wong K.S., Dong F., Telatar M., Lorch J.H., Alexander E.K., Marqusee E., Cho N.L., Nehs M.A., Doherty G.M., Afkhami M. (2021). Papillary Thyroid Carcinoma with High-Grade Features Versus Poorly Differentiated Thyroid Carcinoma: An Analysis of Clinicopathologic and Molecular Features and Outcome. Thyroid.

[13] Jannin A., Escande A., Al Ghuzlan A., Blanchard P., Hartl D., Chevalier B., Deschamps F., Lamartina L., Lacroix L., Dupuy C. (2022). Anaplastic Thyroid Carcinoma: An Update. Cancers.

[14] Ibrahimpasic T., Ghossein R., Shah J.P., Ganly I. (2019). Poorly Differentiated Carcinoma of the Thyroid Gland: Current Status and Future Prospects. Thyroid.

[15] Yu A.C., Han A.Y., Cronkite D.A., Sajed D., St John M.A. (2023). Anaplastic Transformation of Differentiated Thyroid Carcinoma. Laryngoscope.

[16] Esmaili J.H., Hafez G.R., Warner T.F. (1983). Anaplastic carcinoma of the thyroid with osteoclast-like giant cells. Cancer.

[17] Kapp D.S., LiVolsi V.A., Sanders M.M. (1982). Anaplastic carcinoma following well-differentiated thyroid cancer: Etiological considerations. Yale J. Biol. Med..

[18] Forma A., Kłodnicka K., Pająk W., Flieger J., Teresińska B., Januszewski J., Baj J. (2025). Thyroid Cancer: Epidemiology, Classification, Risk Factors, Diagnostic and Prognostic Markers, and Current Treatment Strategies. Int. J. Mol. Sci..

[19] Prete A., Borges de Souza P., Censi S., Muzza M., Nucci N., Sponziello M. (2020). Update on Fundamental Mechanisms of Thyroid Cancer. Front. Endocrinol..

[20] Bronner M.P., LiVolsi V.A. (1991). Spindle cell squamous carcinoma of the thyroid: An unusual anaplastic tumor associated with tall cell papillary cancer. Mod. Pathol..

[21] Van der Laan B.F.A.M., Freeman J.L., Tsanq R.W., Asa S.L. (1993). The association of well-differentiated thyroid carcinoma with insular or anaplastic thyroid carcinoma; evidence for dedifferentiation in tumor progression. Endocr. Pathol..

[22] Volante M., Collini P., Nikiforov Y.E., Sakamoto A., Kakudo K., Katoh R., Lloyd R.V., LiVolsi V.A., Papotti M., Sobrinho-Simoes M. (2007). Poorly differentiated thyroid carcinoma: The Turin proposal for the use of uniform diagnostic criteria and an algorithmic diagnostic approach. Am. J. Surg. Pathol..

[23] World Health Organization Classification of Tumors Editorial Board (2017). WHO Classification of Tumors of Endocrine Organs.

[24] World Health Organization Classification of Tumors Editorial Board (2022). WHO Classification of Tumors: Endocrine and Neuroendocrine Tumors.

[25] Yukino K., Komohara Y., Zhao S., Yamada R., Fujiwara Y., Murakami A., Shimoda Y., Saito H., Orita Y. (2025). Anaplastic thyroid carcinoma with osteoclast-like giant cells: A case report and a study of a potential therapeutic approach. Med. Mol. Morphol..

[26] Nguyen D., Htun N.N., Wang B., Lee B., Johnson C. (2023). An Anaplastic Thyroid Carcinoma of the Giant-Cell Type from a Mediastinal Ectopic Thyroid Gland. Diagnostics.

[27] Gaumann A., Hansen T., Köhler H.H., Kommoss F., Mann W., Maurer J., Kirkpatrick C.J., Kriegsmann J. (2001). The expression of cathepsins in osteoclast-like giant cells of an anaplastic thyroid carcinoma with tracheal perforation. Pathol. Res. Pract..

[28] Bernstein J., Virk R.K., Hui P., Prasad A., Westra W.H., Tallini G., Adeniran A.J., Udelsman R., Sasaki C.T., Roman S.A. (2013). Tall cell variant of papillary thyroid microcarcinoma: Clinicopathologic features with BRAF(V600E) mutational analysis. Thyroid.

[29] Zou M., Baitei E.Y., Alzahrani A.S., BinHumaid F.S., Alkhafaji D., Al-Rijjal R.A., Meyer B.F., Shi Y. (2014). Concomitant RAS, RET/PTC, or BRAF mutations in advanced stage of papillary thyroid carcinoma. Thyroid.

[30] Alhejaily A.G., Alhuzaim O., Aljohani N., Alghamdi D., Salem R.O. (2025). Role of *TERT* gene mutation in the pathogenesis of anaplastic thyroid carcinoma (Review). Mol. Clin. Oncol..

[31] Quiros R.M., Ding H.G., Gattuso P., Prinz R.A., Xu X. (2005). Evidence that one subset of anaplastic thyroid carcinomas are derived from papillary carcinomas due to BRAF and p53 mutations. Cancer.

[32] García-Rostán G., Costa A.M., Pereira-Castro I., Salvatore G., Hernandez R., Hermsem M.J., Herrero A., Fusco A., Cameselle-Teijeiro J., Santoro M. (2005). Mutation of the PIK3CA gene in anaplastic thyroid cancer. Cancer Res..

[33] Sasanakietkul T., Murtha T.D., Javid M., Korah R., Carling T. (2018). Epigenetic modifications in poorly differentiated and anaplastic thyroid cancer. Mol. Cell. Endocrinol..

[34] Shakib H., Rajabi S., Dehghan M.H., Mashayekhi F.J., Safari-Alighiarloo N., Hedayati M. (2019). Epithelial-to-mesenchymal transition in thyroid cancer: A comprehensive review. Endocrine.

[35] Fuziwara C.S., Saito K.C., Kimura E.T. (2020). Thyroid Follicular Cell Loss of Differentiation Induced by MicroRNA miR-17-92 Cluster Is Attenuated by CRISPR/Cas9n Gene Silencing in Anaplastic Thyroid Cancer. Thyroid.

[36] Zeng P.Y.F., Prokopec S.D., Lai S.Y., Pinto N., Chan-Seng-Yue M.A., Clifton-Bligh R., Williams M.D., Howlett C.J., Plantinga P., Cecchini M.J. (2024). The genomic and evolutionary landscapes of anaplastic thyroid carcinoma. Cell Rep..

[37] Busaidy N.L., Konda B., Wei L., Wirth L.J., Devine C., Daniels G.A., DeSouza J.A., Poi M., Seligson N.D., Cabanillas M.E. (2022). Dabrafenib Versus Dabrafenib + Trametinib in *BRAF*-Mutated Radioactive Iodine Refractory Differentiated Thyroid Cancer: Results of a Randomized, Phase 2, Open-Label Multicenter Trial. Thyroid.

